# Thinking and Working

**Published:** 1886-10

**Authors:** 


### Thinking and Working.

The *Popular Science News* tells us that in our present system of education—now happily passing away for a better one—we want one man to be always thinking, and another to be always working; and we call the one a gentleman and the other an operative; whereas the workman ought often to be thinking, and the thinker often to be working, and both should be gentlemen in the best sense. As it is, we make both ungentle, the one envying, the other despising, the other; and the mass of society is made up of morbid unhealthy thinkers and miserable workers. It is only by labor that thought can be made happy; and the profession should be liberal, and there should be less pride felt in peculiarity of employment, and more in the excellence of achievement.

